# Host cell transcriptomic response to the multidrug-resistant *Mycobacterium tuberculosis* clonal outbreak Beijing strain reveals its pathogenic features

**DOI:** 10.1080/21505594.2022.2135268

**Published:** 2022-10-15

**Authors:** Pinidphon Prombutara, Tegar Adriansyah Putra Siregar, Thanida Laopanupong, Phongthon Kanjanasirirat, Tanawadee Khumpanied, Suparerk Borwornpinyo, Awantika Rai, Angkana Chaiprasert, Prasit Palittapongarnpim, Marisa Ponpuak

**Affiliations:** a Omics Sciences and Bioinformatics Center, Faculty of Science, Chulalongkorn University, Bangkok, Thailand; b Microbiome Research Unit for Probiotics in Food and Cosmetics, Faculty of Sciences, Chulalongkorn University, Bangkok, Thailand; c Department of Microbiology, Faculty of Science, Mahidol University, Bangkok, Thailand; d Department of Microbiology, Faculty of Medicine, University of Muhammadiyah Sumatera Utara, Medan, Indonesia; e Excellent Center for Drug Discovery, Faculty of Science, Mahidol University, Bangkok, Thailand; f Department of Biotechnology, Faculty of Science, Mahidol University, Bangkok, Thailand; g School of Biotechnology, KIIT University, Bhubaneswar, India; h Drug-Resistance Tuberculosis Research Fund, Siriraj Foundation, Faculty of Medicine Siriraj Hospital, Mahidol University, Bangkok, Thailand; i Department of Microbiology, Faculty of Medicine Siriraj Hospital, Mahidol University, Bangkok, Thailand; j Pornchai Matangkasombut Center for Microbial Genomics, Department of Microbiology, Faculty of Science, Mahidol University, Bangkok, Thailand; k National Center for Genetic Engineering and Biotechnology, National Science and Technology Development Agency, Pratumthani, Thailand

**Keywords:** *Mycobacterium tuberculosis*, drug resistance, RNA-Seq, Beijing strain, Tuberculosis

## Abstract

The upsurge of multidrug-resistant infections has rendered tuberculosis the principal cause of death among infectious diseases. A clonal outbreak multidrug-resistant triggering strain of *Mycobacterium tuberculosis* was identified in Kanchanaburi Province, labelled “MKR superspreader,” which was found to subsequently spread to other regions, as revealed by prior epidemiological reports in Thailand. Herein, we showed that the MKR displayed a higher growth rate upon infection into host macrophages in comparison with the H37Rv reference strain. To further elucidate MKR’s biology, we utilized RNA-Seq and differential gene expression analyses to identify host factors involved in the intracellular viability of the MKR. A set of host genes function in the cellular response to lipid pathway was found to be uniquely up-regulated in host macrophages infected with the MKR, but not those infected with H37Rv. Within this set of genes, the IL-36 cytokines which regulate host cell cholesterol metabolism and resistance against mycobacteria attracted our interest, as our previous study revealed that the MKR elevated genes associated with cholesterol breakdown during its growth inside host macrophages. Indeed, when comparing macrophages infected with the MKR to H37Rv-infected cells, our RNA-Seq data showed that the expression ratio of *IL-36RN*, the negative regulator of the IL-36 pathway, to that of *IL-36G* was greater in macrophages infected with the MKR. Furthermore, the MKR’s intracellular survival and increased intracellular cholesterol level in the MKR-infected macrophages were diminished with decreased *IL-36RN* expression. Overall, our results indicated that *IL-36RN* could serve as a new target against this emerging multidrug-resistant *M. tuberculosis* strain.

## Introduction

Tuberculosis (TB) remains a prominent cause of mortality, with 1.5 million deaths reported in 2021 stemming from a single infectious agent [1]. Globally, TB control programs have been threatened by the emergence of multidrug-resistant (MDR) and extensively drug-resistant (XDR) *M. tuberculosis* (Mtb) strains [1]. Among the various Mtb lineages, the modern Beijing family is recognized as a very efficacious genotype for its pervasive propagation across regions correlated with large outbreaks and multidrug resistance [2,3]. In addition, previous studies demonstrated that the Beijing strains possess a superior ability to endure inside host macrophages with elevated mycobacterial loads and mortality rates in animal models in addition to initiate high acid-fast bacilli smear-positive sputum in patients [4–9]. Nevertheless, the factors responsible for their enhanced survival inside hosts remain unclear.

After entering into human lungs, Mtb is phagocytosed and resides primarily within the alveolar macrophages [10]. Subsequent granuloma formation can then control Mtb growth within the host macrophages in immunocompetent individuals. Consequently, the infection becomes latent with a 10% probability that the mycobacteria will reactivate triggering active TB disease later in life [11]. Nevertheless, active TB disease progresses immediately in approximately 10% of infected persons due to the weak immune systems of the hosts. The mechanisms of Mtb reactivation and causes of active TB disease remain unspecified. Nevertheless, the mycobacterial growth spurt inside the host macrophages is thought to bring about the necrotic death of host cells [12], thus discharging the mycobacteria into the extracellular environment, which promotes further growth, strong inflammation, dissemination and transmission [12–14]. Consequently, the ability to grow intracellularly will determine the number of Mtb inside the host cells, which will subsequently dictate the number of extracellular Mtb within the infectious droplet nuclei ready to be transmitted to uninfected people. Thus, the increased intracellular growth capacity of the Mtb is assumed to correlate with its increased transmission.

Previous MDR-TB epidemiological analyses in Thailand uncovered a substantial outbreak in Kanchanaburi Province [3,15]. The outbreak causing strain named “MKR superspreader,” belongs to the modern Beijing genotype sequence type 10, which is exceedingly widespread in numerous countries [16–18]. More specifically, the MKR superspreader belongs to sublineage L2.2.M3 (Asian African 3) [19], which are found to expand in other areas as well [20,21]. The widespread of this emerging MDR-TB causing strain suggests the superior capability of the MKR superspreader to propagate inside the host. In our previous study, we carried out a whole transcriptomic analysis and identified the mycobacterial factors required for the growth of the MKR inside the host macrophages, which include gene function in the cholesterol breakdown and ESX-1 secretion system [22]. However, the host factors involved in the intracellular viability of MKR remain undetermined.

In an attempt to identify the host factors regulating the intracellular growth of MKR inside the host macrophages, herein we conducted RNA-Seq analyses of host cells infected with the MKR superspreader versus those infected by the Mtb reference strain H37Rv. We discovered that the MKR-infected host macrophages uniquely up-regulated genes functioning in the cellular response to lipid pathways, while those infected with H37Rv did not. In addition, the network analyses showed that the differentially expressed genes (DEGs) within the cellular response to lipid pathway interacted with the response to bacteria pathways. As our previous study revealed that the MKR upregulated its gene function in cholesterol degradation during growth inside host macrophages, the IL-36 cytokines previously demonstrated to play a role in host cell cholesterol metabolism and defence against Mtb [23] attracted our attention. Our RNA-Seq data showed a significant increase in the expression ratio of *IL-36RN*, the negative regulator of the IL-36 pathway, over that of *IL-36G* in the host macrophages infected with the MKR, compared with the H37Rv-infected cells. The results were verified by qRT-PCR. Moreover, *IL-36RN* depletion resulted in the decreased intracellular survival of the MKR and a reduction in the intracellular cholesterol level of the MKR-infected cells. Our results suggest that IL-36RN plays a crucial role in the intracellular viability of the MKR, and consequently may be targeted for drug discovery against this emerging multidrug-resistant *M. tuberculosis* strain.

## Results

### Increased intracellular growth of MKR inside host macrophages but not that of H37Rv

**Figure 1.**
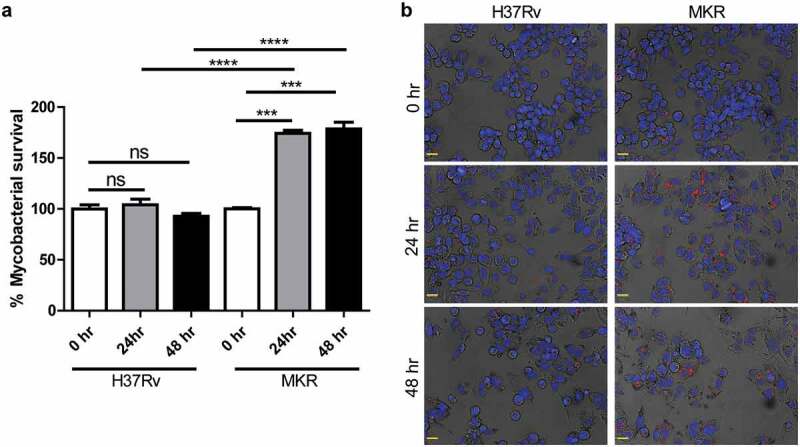
Intracellular survival rates of the MKR superspreader and H37Rv inside host macrophages.(a-b) THP-1 cells were infected with mCherry-expressing MKR superspreader or H37Rv for 1 hr. Cells were washed to eliminate the uninternalized mycobacteria. At 0, 24, and 48 hr after infection, cells were fixed and processed for high-content image analysis to determine intracellular mycobacterial number per cell. Percent mycobacterial survival was calculated and then compared (a). Representative images are shown in (b). Data are means ± SEM from at least three independent experiments; ns, non-significant, ***p < 0.001 and ****p < 0.0001, all relative to the 0 hr control set to 100%, were determined by one-way ANOVA with Tukey’s multiple comparison test. Bar 20 μm.

As prior epidemiological reports in Thailand have shown that the multidrug-resistant Mtb clonal outbreak Beijing strain MKR superspreader was prevalent [3,15,21], we hypothesized that the MKR might have an enhanced intracellular growth capacity inside the host macrophages. We then determined the MKR’s intracellular growth by infecting human macrophage-differentiated THP-1 cells with the mCherry-expressing MKR or H37Rv at MOI of 10 for 1 hr. Cells were subsequently washed to get rid of the uninternalized mycobacteria. At 0, 24, and 48 hr after infection, cells were fixed and processed for high-content image analysis to determine the number of intracellular Mtb. The results showed a significant increase in the MKR’s intracellular growth at 24 and 48 hr after infection, while such effects were not observed with that of H37Rv (Figures 1a,b). The enhanced intracellular growth of the MKR when compared to that of H37Rv was also confirmed by CFU analysis (Supplementary Figure S1a). In addition, we determined the MKR’s ability to enter into host macrophages and found a slight but significant decrease in the MKR’s internalized rate when compared to that of H37Rv (Supplementary Figure S1b-c). Moreover, the in vitro growth rate of the MKR appeared to be slower than that of H37Rv (Supplementary Figure S2). These findings suggest that, upon entering into host cells, the MKR has an increased capability to grow intracellularly when compared to the reference strain H37Rv.

### RNA-Seq and differential gene expression analyses

**Figure 2.**
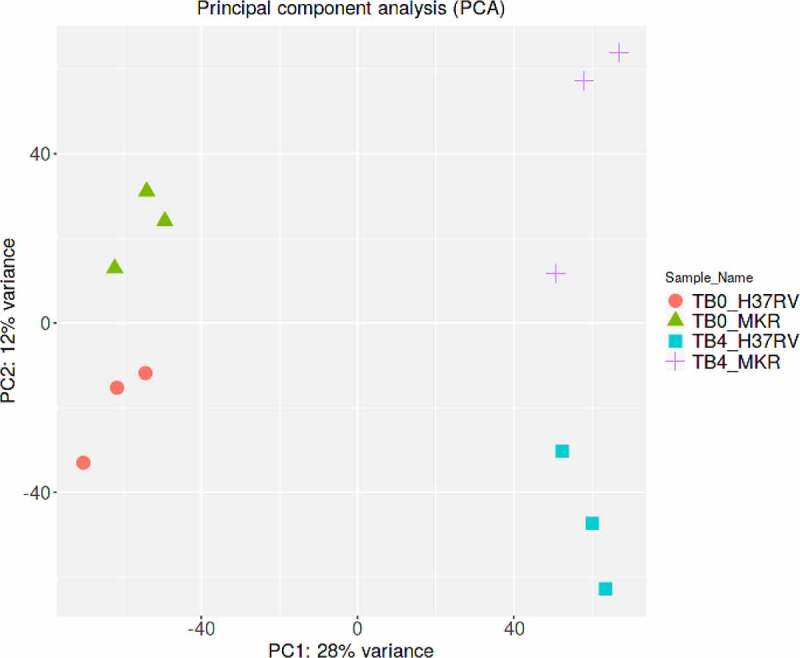
Principle component analysis. Samples from the same experimental conditions appear to cluster together in accordance with their gene expression profiles. The first principal component (PC1) captures the gene expression profile differences across the 2 time points post-infection (0 and 4 hr post-infection), while the second principal component (PC2) captures the gene expression profile differences among H37Rv- and MKR-infected macrophages. TB0_H37RV, macrophages infected with H37Rv at 0 hr post-infection; TB4_H37RV, macrophages infected with H37Rv at 4 hr post-infection; TB0_MKR, macrophages infected with MKR at 0 hr post-infection; TB4_MKR, macrophages infected with MKR at 4 hr post-infection.

**Figure 3.**
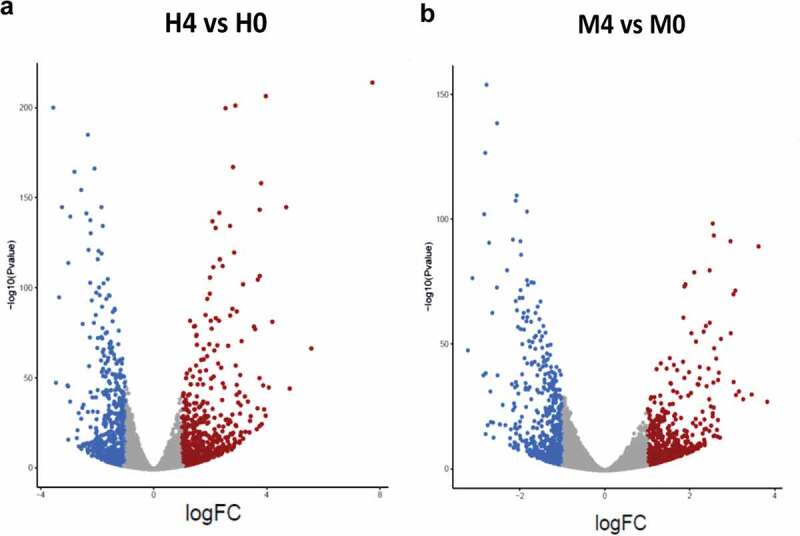
Differential gene expression analyses. (a-b) Volcano plots were used to determine the significantly different expressed transcripts among biological conditions (experimental group versus baseline): H4 vs H0 (a) and M4 vs M0 (b). Only log_2_(fold change) ≥ 2 or ≤ 2 and false discovery rate (FDR) adjusted p-values < 0.05 were analyzed. The upregulated transcripts were labelled in red and downregulated transcripts were labelled in blue. H0, macrophages infected with H37Rv at 0 hr post-infection; H4, macrophages infected with H37Rv at 4 hr post-infection; M0, macrophages infected with MKR at 0 hr post-infection; M4, macrophages infected with MKR at 4 hr post-infection.

To identify the host factors contributing to the intracellular growth of MKR, RNA-Seq and differential gene expression analyses were performed on host macrophages infected with the MKR versus those infected by H37Rv to compare the response at 0 hr to that of 4 hr post-infection (T0 vs T4). RNA-Seq data derived from the uninfected macrophages at 0 hr and 4 hr post-infection were used as controls. Three biological replicates were performed and a mean of 40.8 ± 8.4 million paired reads per individual library were detected. The total reads of 78.3 ± 3.9% were mapped into the human reference genome GRCh38. The principal component analysis (PCA) plot showed that the samples were clustered by their biological replicates (Figure 2). Subsequently, gene expression transcripts were counted and normalized for the differential gene expression analysis using DESeq2. RNA-Seq comparisons of the 2 infected time points revealed transcriptome profiles, in which 5,194 and 4,855 genes were differentially expressed in the host cells infected with H37Rv and MKR, respectively (fragments per kilobase of transcript per million mapped reads (FPKM) > 10 per sample and a false discovery rate (FDR) < 0.05) (Figures 3a,b; Supplementary Table S1). Among these, the expressions of 2,321 genes were increased, while those of 2,873 genes were decreased in the H37Rv-infected macrophages. In the MKR-infected macrophages, the expressions of 2,103 genes were increased, while 2,752 genes were decreased in their expressions.

### qRT-PCR confirmation

**Figure 4.**
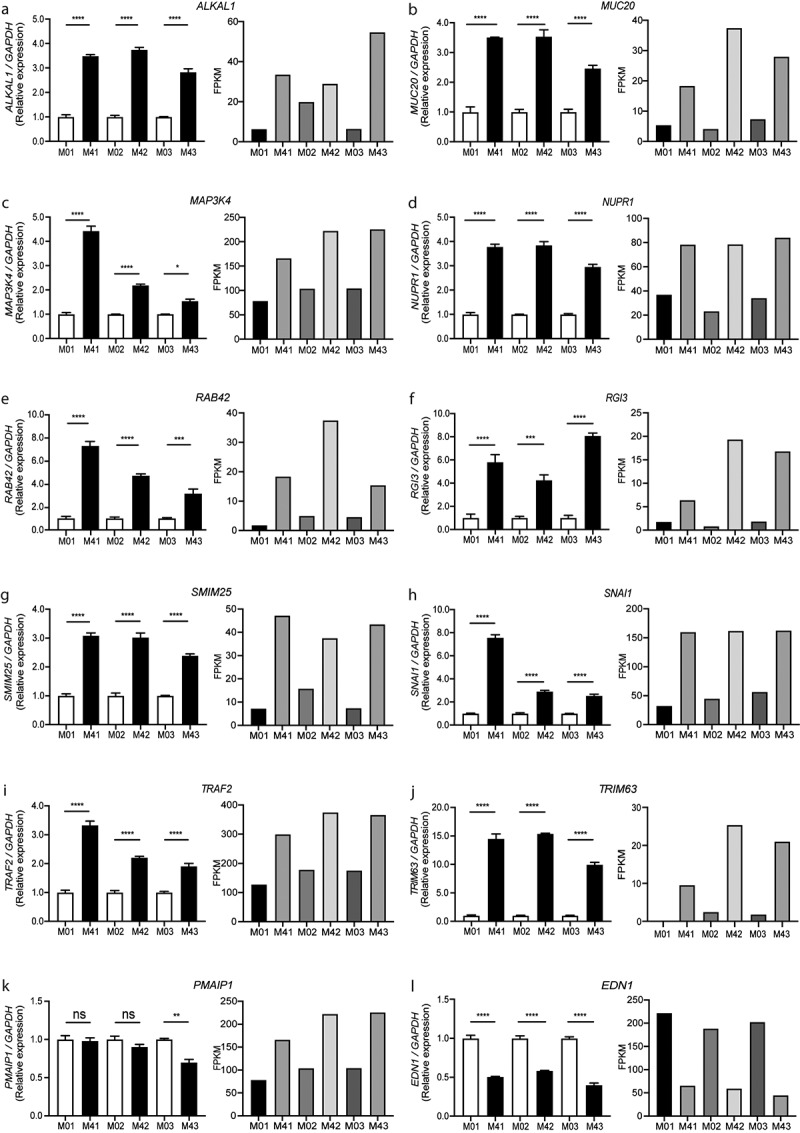
Validation of twelve DEGs in the MKR-infected macrophages by qRT-PCR. (a-l) THP-1 macrophages were infected with H37Rv or MKR at MOI 10 for 1hr and samples were analyzed at either 0 hr or 4 hr post-infection. Total RNAs were isolated and cDNAs were synthesized. The expression levels of twelve genes shown to be differentially expressed by the RNA-Seq data were quantified by qRT-PCR. 2^−∆∆ct^ was used for normalization and relative quantification. Data are the means ± SEM from three independent experiments; ns, non-significant, *p < 0.05, **p < 0.01,***p < 0.001 and ****p < 0.0001, all relative to the respective 0 hr control set to 1.0 were determined by one-way ANOVA with Tukey’s multiple comparison test. M0, macrophages infected with MKR at 0 hr post-infection; M4, macrophages infected with MKR at 4 hr post-infection; M01, M02, M03 correspond to three biological repeats of macrophages infected with MKR at 0 hr post-infection; M41, M42, M43 correspond to three biological repeats of macrophages infected with MKR at 4 hr post-infection; FPKM, fragments per kilobase of transcript per million mapped reads.

Verification of the RNA-Seq results was conducted through qRT-PCR analyses, for which a set of 12 significantly DEGs in the host macrophages in response to MKR infection were validated (Figure 4a–l). Three technical replicates for each of the three biological replicates were tested for gene expression alteration. For relative quantification, the transcription levels of genes in the MKR-infected host macrophages at 4 hr post-infection were compared with their respective host cells at 0 hr post-infection control and normalized to the *GAPDH* gene. The 11 genes that were significantly up-regulated in the host macrophages infected with the MKR at 4 hr post-infection compared with that of 0 hr post-infection chosen for the qRT-PCR analyses were *ALKAL1*, *MUC20*, *MAP3K4*, *NUPR1*, *RAB42*, *RGI3*, *SMIM25*, *SNAI1*, *TRAF2*, *TRIM63*, and *PMAIP1*. Additionally, one significantly down-regulated gene in the MKR-infected macrophages at 4 hr versus that of 0 hr post-infection, *EDN1*, from the same differential gene expression analysis, was selected. The results showed that the qRT-PCR relative quantifications obtained from 11 out of 12 genes (with the exception of *PMAIP1*) were consistent with our RNA-Seq transcript expression data.

### Functional enrichment and network analyses

**Figure 5.**
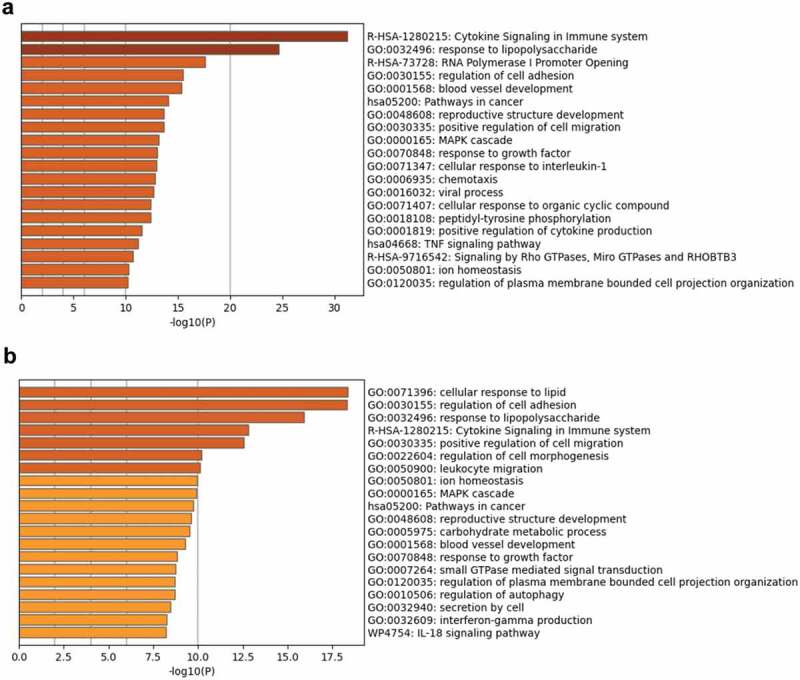
Functional enrichment analysis of the upregulated DEGs in the H37Rv- and MKR-infected macrophages was performed using Metascape.(a) Bar graph showing the top 20 enriched ontology clusters of the H37Rv-infected macrophages. (b) Bar graph showing the top 20 enriched ontology clusters of the MKR-infected macrophages. The enriched ontology clusters were ranked by -log10 p-value of gene enrichment.

To determine the factors involved in regulating the growth of MKR inside the host macrophages, functional enrichment and network analyses were conducted. Ontology cluster assignments were used to determine and compare the potential functions of the upregulated DEGs between H37Rv- and MKR-infected macrophages at 4 hr versus that of 0 hr post-infection (Figures 5a,b). The results showed 11 ontology clusters: the cytokine signalling in the immune system, response to lipopolysaccharide, regulation of cell adhesion, positive regulation of cell migration, ion homoeostasis, MAPK cascade, pathways in cancer, reproductive structure development, blood vessel development, response to growth factor, and regulation of plasma membrane-bounded cell projection organization, to be enriched in both H37Rv- and MKR-infected macrophages. However, the results showed the enrichment of ontology clusters, which included the cellular response to lipid, regulation of cell morphogenesis, leukocyte migration, carbohydrate metabolic process, small GTPase mediated signal transduction, regulation of autophagy, secretion by cells, interferon-gamma production, and IL-18 signalling pathway to be uniquely upregulated in the MKR-infected macrophages at 4 hr versus that of 0 hr post-infection, but not in cells infected with H37Rv.

**Figure 6.**
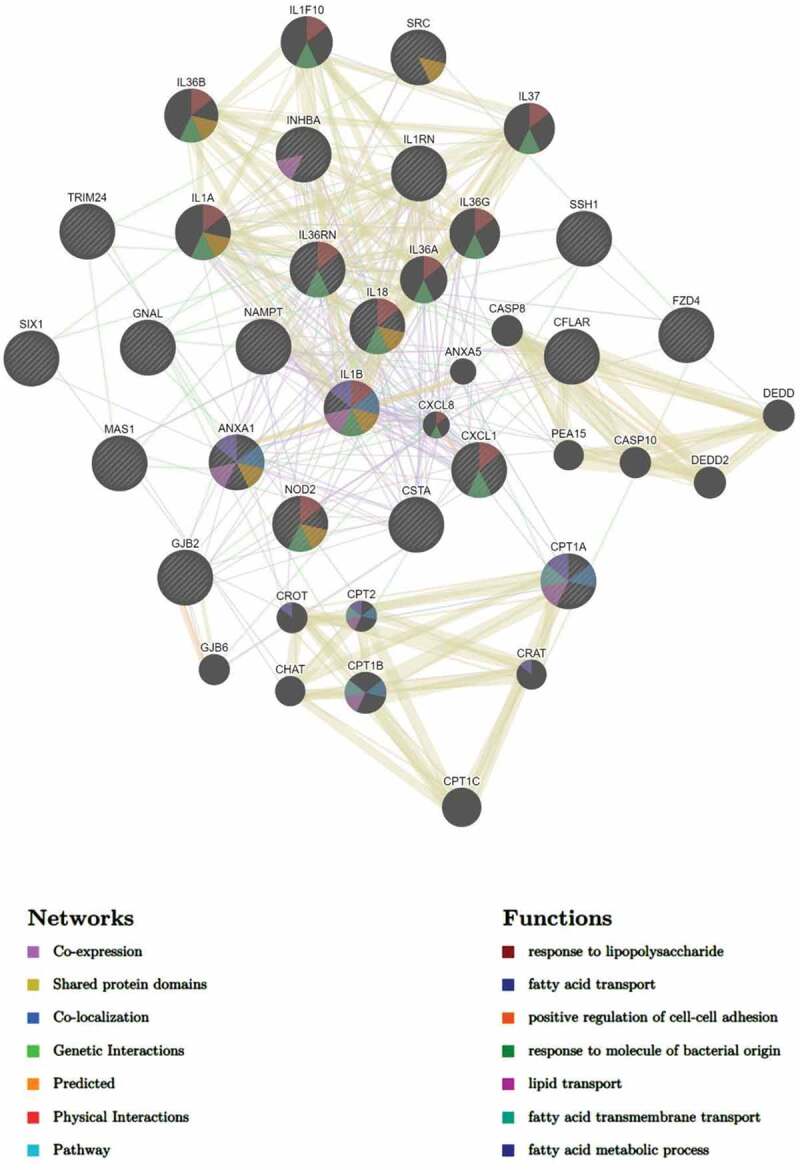
DEGs of the MKR-infected macrophages that function in the cellular response to the lipid pathway were subjected to network analysis using GeneMANIA. The network interactions identified included the fatty acid transport, lipid transport, and response to molecule of bacterial origin, as well as response to lipopolysaccharide, positive regulation of cell-cell adhesion, fatty acid transmembrane transport, and the fatty acid metabolic process.

A network of interactions associated with the most significantly upregulated pathway in the MKR-infected macrophages, the cellular response to lipid pathway (Figure 5b), were analysed as well. To determine this, we subjected the DEGs in the cellular response to lipid pathway to GeneMANIA to identify any possible network interactions based on co-expression, shared protein domains, co-localization, genetic interactions, predicted, physical interactions, and pathway (Figure 6). The results showed that the DEGs in the cellular response to lipid pathway exhibited network interactions to the fatty acid transport, lipid transport, fatty acid transmembrane transport, and fatty acid metabolic process pathways. Interestingly, the DEGs in the cellular response to lipid pathway also exhibited network interactions to the cellular response to bacteria pathways, which included the response to lipopolysaccharide and response to molecule of bacterial origin pathways. These interacted genes included *CXCL1*, *CXCL8*, *IL-36A*, *IL-36B*, *IL-36G, IL-36RN*, *IL-37*, *IL-1F10*, *IL-1A*, *IL-1B*, *IL-18* and *NOD2*. As our recent study showed that the MKR up-regulates genes functioning in the cholesterol breakdown during its growth inside the host macrophages and this pathway is crucial for the MKR’s intracellular survival [22], the IL-36 cytokines previously shown to play role in host cell cholesterol metabolism and defence against Mtb [23] attracted our interest and were investigated further.

### The ratio of IL-36RN/IL-36G expression is increased in the MKR-infected macrophages when compared to that of the H37Rv-infected cells

The IL-36 signalling pathway comprises the IL-36 receptor (encoded by the *IL-36 R* gene), the pro-inflammatory cytokines IL-36α, IL-36β, and IL-36γ (encoded by the *IL-36A*, *IL-36B*, and *IL-36G* genes, respectively), and the IL-36 receptor antagonist IL-36Ra (encoded by the *IL-36RN* gene) [24]. The IL-36 pro-inflammatory cytokines were previously demonstrated to function in host defence against *M. bovis* BCG and Mtb [25]. Specifically, IL-36γ was shown to regulate host cell cholesterol metabolism and Mtb growth restriction in host macrophages [23,26]. On the other hand, IL-36Ra is a secreted molecule that can counteract the inflammatory effects of the IL-36 pro-inflammatory cytokines upon binding to the IL-36 receptor [27].

**Figure 7.**
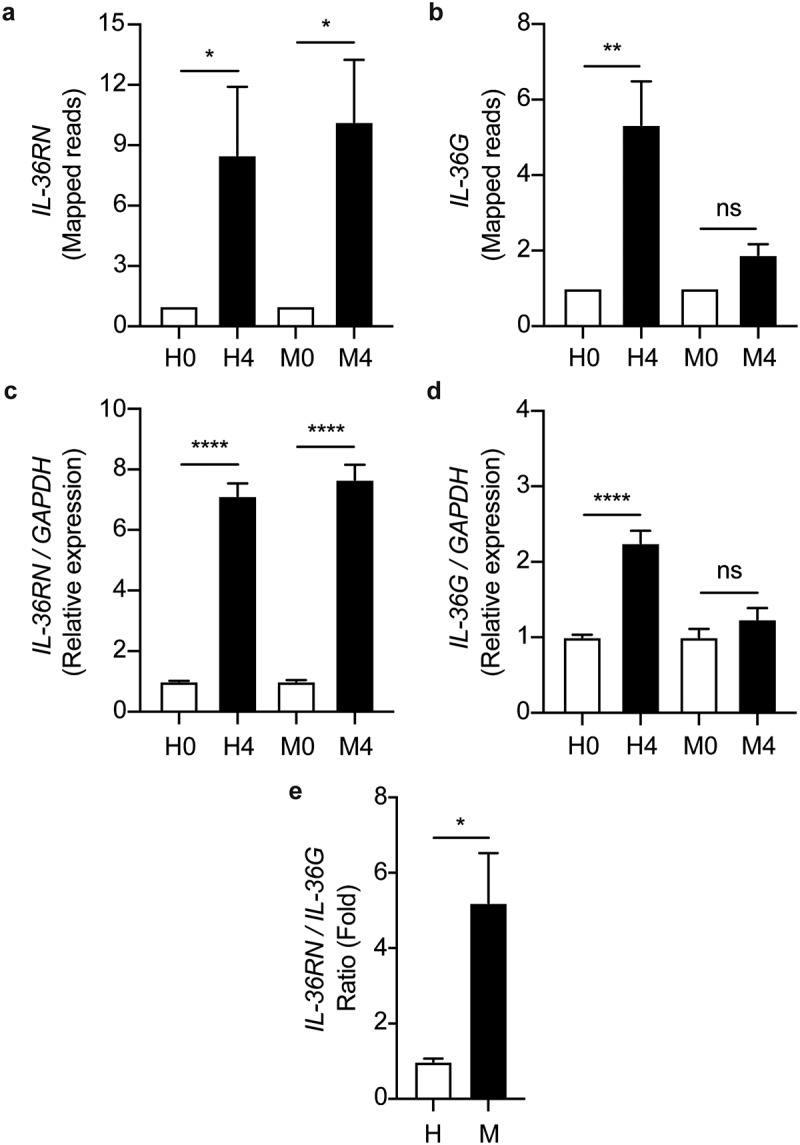
*IL-36RN*/*IL-36G* expression ratio is increased in the MKR-infected macrophages when compared to those infected with H37Rv. (a-b) THP-1 macrophages were infected with H37Rv or MKR at MOI 10 for 1 hr and samples were analyzed at either 0 hr or 4 hr post-infection. The expression levels of *IL-36RN*(a) or *IL-36G* (b) transcripts were determined by RNA-Seq and presented as mapped reads. Data are means ± SEM from three independent experiments; ns, non-significant, *p < 0.05 and **p < 0.01, relative to the respective 0 hr control set of 1.0 were determined by one-way ANOVA with Tukey’s multiple comparison test. (c-d) The expressions of *IL-36RN*(c) or *IL-36G* (d) were then confirmed by qRT-PCR. 2^−∆∆ct^ was used for normalization and relative quantification. Data are means ± SEM from at least three independent experiments; ns, non-significant and ****p < 0.0001, relative to the respective 0 hr control set of 1.0 were determined by one-way ANOVA with Tukey’s multiple comparison test. (e)The *IL-36RN/IL-36G* expression ratio of H37Rv- or MKR-infected macrophages was determined by dividing the respective normalized RNA-Seq mapped reads. *p < 0.05, relative to the H37Rv control set to 1.0 was determined by Student’s t-test. H0, macrophages infected with H37Rv at 0 hr post-infection; H4, macrophages infected with H37Rv at 4 hr post-infection; M0, macrophages infected with MKR at 0 hr post-infection; M4, macrophages infected with MKR at 4 hr post-infection.

Based on the ontology cluster enrichment and network interaction analyses mentioned above (Figure 5b and 6), we then investigated the possible role of the IL-36 pathway in the intracellular growth regulation of the MKR inside the host macrophages. We looked into our RNA-Seq data for the expression changes of the IL-36 cytokines in host macrophages in response to the MKR infection. The expressions of *IL-36A* and *IL-36B* were not significantly different in either the H37Rv- or MKR-infected macrophages. Interestingly, we found that while the expressions of *IL-36RN*, encoding the negative regulator of the IL-36 pathway IL-36Ra, were significantly upregulated in both the H37Rv- and MKR-infected macrophages, the expression of *IL-36G* was found to be significantly augmented in the H37Rv-infected but not MKR-infected cells (Figure 7a,b). These results were verified by qRT-PCR (Figure 7c,d). Consequently, the expression ratio of *IL-36RN* over that of *IL-36G* was significantly higher in the MKR-infected macrophages when compared to those infected with H37Rv (Figure 7e). The data suggested that the increase in the *IL-36RN*/*IL-36G* expression ratio might contribute to the enhanced intracellular survival of the MKR.

### IL-36RN is important for the MKR’s intracellular survival

**Figure 8.**
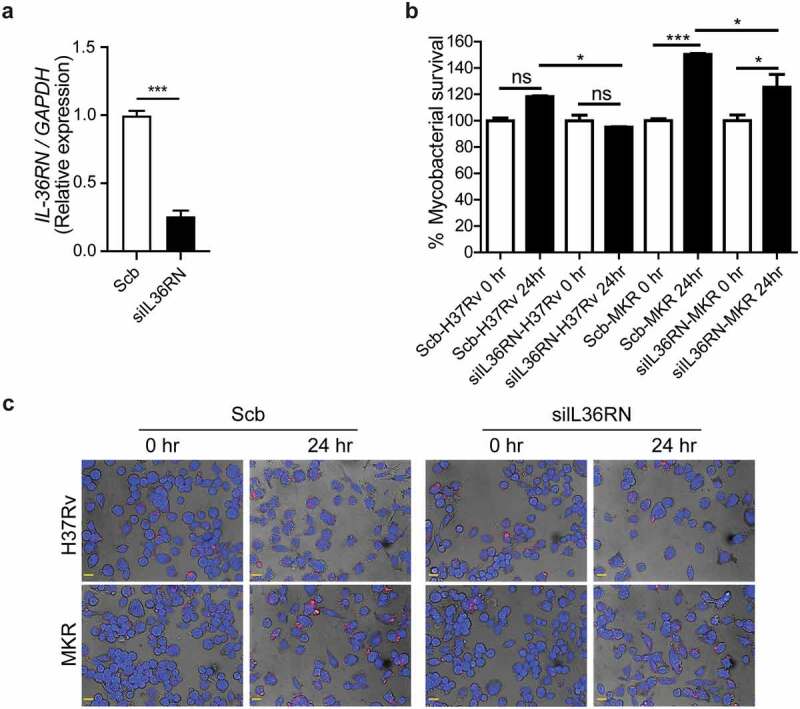
*IL-36RN* is crucial for the intracellular viability of MKR. (a)THP-1 cells were transfected with the non-targeted scrambled control (Scb) or *IL-36RN*-targeted siRNAs. The *IL-36RN* expression levels were then determined by qRT-PCR at 48 hr post-transfection. Data are means ± SEM from three independent experiments; ***p < 0.001, relative to Scb control set to 1.0 was determined by Student’s t-test. (b-c) siRNA-transfected THP-1 cells were infected with mCherry-expressing H37Rv or MKR for 1 hr and washed with complete media to get rid of the uninternalized mycobacteria. Cells were then further incubated in the complete media for 24 hr and subsequently processed for high-content image analysis to determine the intracellular mycobacterial number per cell. Percent mycobacterial survival was calculated and then compared (b). Representative images are shown in (c). Data are means ± SEM from at least three independent experiments; ns, non-significant, *p<0.05 and ***p < 0.001, all relative to the respective 0 hr control set to 100%, were determined by one-way ANOVA with Tukey’s multiple comparison test. Bar 20 μm.

To determine whether the intracellular growth of the MKR could be regulated by the *IL-36RN*/*IL-36G* expression ratio, we depleted the *IL-36RN* expression from human the THP-1 cells using siRNA-knockdown technology. Successful knockdown was verified by qRT-PCR (Figure 8a). Cells were then infected with the mCherry-expressing MKR or H37Rv at MOI of 10 for 1 hr, washed to eliminate the uninternalized mycobacteria, and processed for high-content image analysis at the indicated time points to determine the number of intracellular Mtb. In agreement with our results in the wild-type macrophages, there was a significant increase in the intracellular growth of MKR inside the scrambled siRNA-transfected control cells at 24 hr post-infection. However, such an increase was not detected in the scrambled siRNA-transfected H37Rv-infected cells (Figure 8b,c). Upon *IL-36RN* depletion, however, a significant decrease in the MKR’s intracellular viability was observed at 24 hr post-infection when compared to that of the scrambled siRNA-transfected control cells (Figure 8b,c). These results were also confirmed by CFU analysis (Supplementary Figure S3a). Of note, we did not detect any difference in the Mtb internalization ability between cells transfected with scrambled control siRNAs and those transfected with siRNAs against *IL-36RN* (Supplementary Figure S3b-c). These findings supported the important role of *IL-36RN* in the intracellular survival of the MKR.

### IL-36RN/IL-36G expression ratio is augmented in the scrambled siRNA-transfected control cells infected with the MKR when compared to those infected with H37Rv and IL-36RN is crucial for the enhanced intracellular cholesterol level in host cells by the MKR

**Figure 9.**
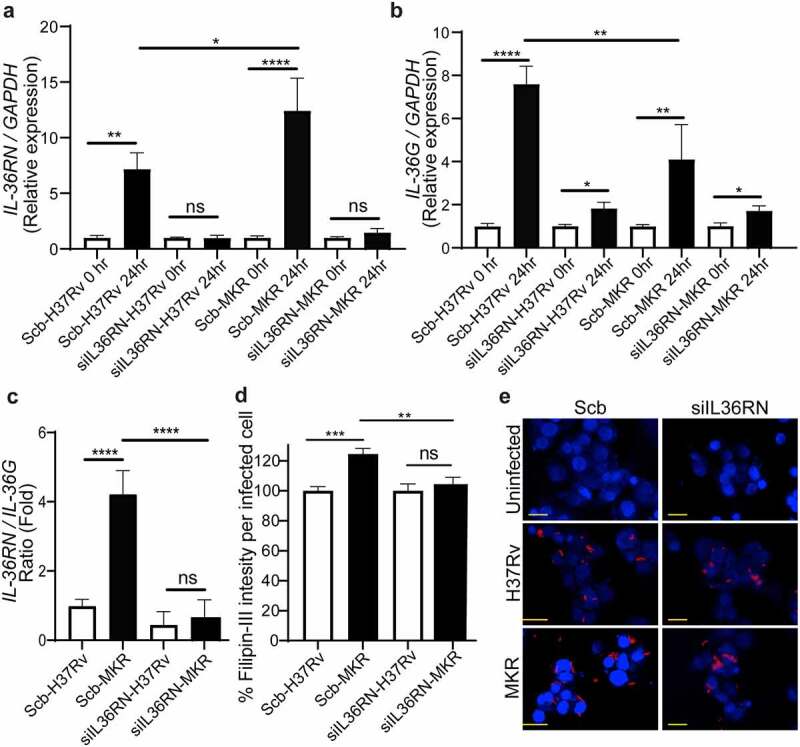
*IL-36RN*/*IL-36G* expression ratio is increased in the scrambled siRNA-transfected MKR-infected macrophages when compared to those infected with H37Rv and *IL-36RN* is important for the increased cholesterol level in host macrophages by the MKR. (a-b) THP-1 cells were transfected with the non-targeted scrambled control (Scb) or *IL-36RN*-targeted siRNAs. At 48 hr post transfection, cells were infected with H37Rv or MKR at MOI 10 for 1 hr and samples were analyzed at either 0 hr or 24 hr post-infection. The expression levels of *IL-36RN*(a) or *IL-36G* (b) transcripts were determined byqRT-PCR. 2^−∆∆ct^ was used for normalization and relative quantification. Data are means ± SEM from at least three independent experiments; ns, non-significant, *p < 0.05, **p < 0.01 and ****p < 0.0001, relative to the respective 0 hr control set of 1.0 were determined by one-way ANOVA with Dunnett’s multiple comparison test. (c)The *IL-36RN/IL-36G* expression ratio of H37Rv- or MKR-infected macrophages transfected with scrambled control or *IL-36RN*-targeted siRNAs were determined by dividing the respective normalized relative expression at 24 hr post-infection from (a-b). ns, non-significant and ****p < 0.0001, relative to the scrambled siRNA-transfected H37Rv-infected control cells set to 1.0 were determined by one-way ANOVA with Tukey’s multiple comparison test. (d-e) THP-1 cells were transfected with the non-targeted scrambled control (Scb) or *IL-36RN*-targeted siRNAs. At 48 hr post transfection, cells were infected with mCherry-expressing H37Rv or MKR at MOI=10 for 1 hr and washed with complete media to get rid of the uninternalized mycobacteria. Cells were then further incubated in the complete media for 24 hr and subsequently processed for high-content image analysis to determine the intracellular cholesterol levels by Filipin-III staining. Percent Filipin-III intensity per infected cell were calculated and then compared (d). Representative images are shown in (e). Data are means ± SEM from at least three independent experiments; ns, non-significant, **p<0.01 and ***p < 0.001, all relative to the respective H37Rv-infected cells set to 100%, were determined by one-way ANOVA with Bonferroni’s multiple comparison test. Bar 20 μm.

As our results in Figure 8 showed the increase in MKR’s intracellular growth inside the scrambled siRNA-transfected control macrophages at 24 hr post-infection but such effect could not be observed when the cells were infected with H37Rv and the MKR’s intracellular viability was decreased upon *IL-36RN* depletion, we then investigated the expressions of *IL-36RN* and *IL-36G* in these cells. We found that, similar to what we observed in the wildtype macrophages, the expressions of *IL-36RN* were significantly increased in both the H37Rv- and MKR-infected scrambled siRNA-transfected macrophages (Figure 9a). In the *IL-36RN*-deficient cells, however, such enhanced *IL-36RN* expression could no longer be detected (Figure 9a). In agreement with our findings observed with the wild-type macrophages, the expression of *IL-36G* was also found to be significantly augmented in the H37Rv-infected scrambled siRNA-transfected cells (Figure 9b). However, we could now detect a significant enhance in *IL-36G* expression when the scrambled siRNA-transfected macrophages were infected with the MKR, albeit less so when compared to that of the H37Rv-infected cells (Figure 9b). Consequently, the expression ratio of *IL-36RN* over that of *IL-36G* was significantly higher in the scrambled siRNA-transfected MKR-infected macrophages when compared to the cells infected with H37Rv (Figure 9c). These data supported that the increase in the *IL-36RN*/*IL-36G* expression ratio contributed to the enhanced MKR’s intracellular survival. As IL-36 cytokines were shown to regulate host cell cholesterol metabolism and Mtb growth inside host macrophages [23,26], we examined the intracellular cholesterol levels of host cells infected with the MKR or H37Rv. The results showed a significant increase in intracellular cholesterol level when scrambled siRNA-transfected control macrophages were infected with the MKR when compared to those infected with H37Rv (Figure 9d,e). Upon *IL-36RN* depletion, however, the augmented intracellular cholesterol level in the MKR-infected macrophages could no longer be observed. The results suggested the important role of *IL-36RN* in the enhanced intracellular cholesterol level in host cells by the MKR.

## Discussion

MDR-TB is a critical threat to TB control programs around the world. Previous epidemiological studies in Kanchanaburi, Thailand, exposed a significant outbreak of MDR-TB, with the causal agent confirmed as a member of the Mtb Beijing ST10 genotype [3,15] or more specifically L2.2.M3 (Asian African 3). This emergent multidrug-resistant Mtb strain was termed an “MKR superspreader,” and was later observed to have spread to other regions as well [21]. The sublineage L2.2.M3 was also found in considerable numbers in Vietnam and China [19]. However, the underlying mechanism for its extensive transmission in the community remains unclear. In this study, we attempted to study the biology of MKR by using RNA-Seq, functional enrichment, and network analyses to identify the host factors involved in the MKR’s intracellular growth inside the host macrophages. Our whole transcriptome analyses determined several significant DEGs which were up- or down-regulated in the macrophages infected with the MKR compared to those infected with the reference strain H37Rv (Figure 2 and 3), verified by qRT-PCR (Figure 4). Functional enrichment analyses of the significantly upregulated DEGs revealed that the cellular response to lipid pathway was the most significantly enriched and unique to the MKR-infected macrophages, but not of those infected with H37Rv, during Mtb intracellular growth (Figure 5). Network analyses of significant DEGs in the cellular response to lipid pathway showed interactions with the response to bacteria pathways (Figure 6) and pointed to the possible involvement of the IL-36 cytokines in the intracellular survival of MKR. As our previous study showed that, during its proliferation inside the host macrophages, the MKR up-regulates gene function in the cholesterol breakdown pathway, which is essential for the intracellular viability of MKR [22], and findings by others have demonstrated the function of IL-36 cytokines in host cell cholesterol metabolism and defence against Mtb [23], further investigation concerning the possible involvement of IL-36 cytokines in the intracellular growth of MKR is warranted. Indeed, significant increases in the *IL-36RN/IL-36G* expression ratio were observed by both RNA-Seq and qRT-PCR analyses in the MKR-infected host cells compared to those infected with H37Rv (Figure 7 and 9). Moreover, the reduced MKR’s intracellular survival and decreased intracellular cholesterol level in the MKR-infected host macrophages upon the depletion of *IL-*36*RN*, which encodes for the IL-36 receptor antagonist called IL-36Ra, supports the importance of this gene in the MKR’s intracellular viability and ability to increase host cell cholesterol (Figure 8 and 9).

Recently, IL-36 cytokines have been described as members of the IL-1 cytokine family comprising a receptor (IL-36 R), three pro-inflammatory cytokines (IL-36α, IL-36β, and IL-36γ), and a receptor antagonist (IL-36Ra) [24]. The IL-36Ra can compete with IL-36α, IL-36β, and IL-36γ for interaction with the IL-36 R, thus neutralizing the inflammatory effects exerted by the pro-inflammatory cytokines [27]. In the context of infections, the IL-36 pro-inflammatory cytokines were recently demonstrated to play a role in host defence against multiple bacteria including *Streptococcus pneumoniae*, *Klebsiella pneumoniae*, *Legionella pneumophila*, *M. bovis* BCG, and Mtb [23,26,28,29]. It was also revealed that the IL-36 signalling resulted in the induction of type 1 immune responses crucial to the control of intracellular pathogens [30,31]. In the case of Mtb, induced production and secretion of IL-36γ was observed upon infection of host macrophages with the Mtb reference strain H37Rv, dependent upon the Toll-like receptor and MyD88 pathways [26]. In addition, IL-36γ/IL-36 R signalling resulted in the induced production of antimicrobial peptides, leading to enhanced H37Rv elimination in the infected macrophages [26]. Furthermore, a study indicated that IL-36γ promoted H37Rv elimination in the infected macrophages through the induction of autophagy [32]. More interestingly, IL-36γ was recently demonstrated to regulate host cell cholesterol metabolism, including the suppression of cholesterol biosynthesis and induction of cholesterol efflux and turnover, leading to the restraint of H37Rv growth in host macrophages [23]. In agreement with the previous findings, there was no increase in the growth rate of H37Rv at 24 and 48 hr post-infection (Figure 1), unlike the MKR, which showed an increased growth rate inside the host macrophages. In addition, our RNA-Seq and qRT-PCR analyses showed that, while the expressions of *IL-36RN* encoding for the IL-36Ra were found to be increased in both H37Rv- and MKR-infected macrophages upon Mtb entry into host cells at 4 hr post-infection, only the expression of *IL-36G* was found to be up-regulated in macrophages infected with H37Rv, but not in the MKR-infected cells (Figure 7). At 24 hr post-infection, increases in *IL-36RN* and *IL-36G* expressions were detected in both H37Rv- and MKR-infected cells, albeit more increase in *IL-36RN* and less increase in *IL-36G* expressions were seen in the MKR-infected macrophages when compared to that of H37Rv-infected cells (Figure 9). This resulted in the increased expression ratio of *IL-36RN*/*IL-36G* in the MKR-infected macrophages (Figure 7 and 9), which could spare the MKR from the IL-36γ-mediated elimination. Indeed, a decrease in the intracellular survival of MKR could be observed upon the depletion of *IL-36RN* (Figure 8). Further investigations are warranted and are topics in our study to determine whether the increased intracellular viability of the MKR is due to the effects of the IL-36Ra on the inhibition of cholesterol efflux and turnover, alleviation of cholesterol biosynthesis suppression, and/or inhibition of autophagy and antimicrobial peptide production. However, as our previous study showed that the MKR up-regulates the genes implicated in cholesterol digestion during its growth inside the host macrophages, and this pathway is crucial for its intracellular survival [22], examining the effects of IL-36Ra on host cell cholesterol, therefore, is of significant interest. Indeed, our preliminary investigation showed that host cell cholesterol level was enhanced in the MKR-infected macrophages when compared to those infected with H37Rv and that such effect was IL-36Ra dependent (Figure 9). Whether this is due to the block in cholesterol efflux, inhibition in cholesterol turnover, or alleviation of cholesterol biosynthesis suppression awaits further examination.

Host cell cholesterol is a preferential nutrient source for Mtb [33]. Recent studies also demonstrate that the synthesis of Mtb virulence lipids, which include PDIM and SLs [34], depends on the cholesterol and fatty acid metabolism [35]. PDIM was revealed to be crucial for Mtb to fight against host cell immune mediators [36–38]. In addition, PDIM was shown to be needed for the Mtb phagosomal escape into the host cell cytosol [39], resulting in the mycobacterial intracellular growth spurt and subsequent necrotic death of host cells, as well as Mtb extracellular growth and transmission [33]. SLs were proven to inhibit the production of TNF-α, a host cytokine significant for granuloma formation and autophagy, and thus the Mtb containment [40]. It might be possible that the increase in the *IL-36RN*/*IL-36G* expression ratio by the MKR in the infected macrophages may provide increased intracellular cholesterol available for the MKR and, together with the upregulation in its gene functioning in the cholesterol breakdown, the MKR thus has an increased ability to enhance its intracellular growth.

As mentioned above, the expression of *IL-36G* was found to be up-regulated in macrophages infected with H37Rv more than those infected with the MKR (Figure 7 and 9). As IL-36γ production is dependent on the binding of different Mtb PAMPs to the Toll-like receptors 2 and 4 and subsequent signalling through MyD88 [25], one could hypothesize that there may be differences in the Mtb PAMPs possessed by H37Rv and MKR, resulting in altered downstream signalling and leading to IL-36γ induction in the H37Rv-infected macrophages higher than those infected with the MKR. These Mtb PAMPs included lipoproteins, lipoarabinomannan, lipomannan, and Hsp65, in which the latter is recognized by Toll-like receptor 4, while the formers are recognized by Toll-like receptor 2 in the host macrophages [26]. Further study will be needed to determine whether there is a difference in Mtb PAMPs between H37Rv and MKR or any alteration in the downstream signalling leading to IL-36γ induction.

Our data showed that a reduced MKR’s intracellular viability was observed upon depletion of *IL-36RN* (Figure 8). It would be interesting to see whether treatment of the MKR-infected macrophages with the recombinant human IL-36γ would also result in the restriction of the MKR. This would suggest a potential targeting of the IL-36 pathway for treatment against the MKR. In addition, our previous study also showed that FDA-approved drugs that alter host cell cholesterol transportation can inhibit the intracellular growth of MKR [22]. These included aripiprazole, which is currently employed for the treatment of depression and bipolar disorders [41], and manidipine, currently utilized for the treatment of hypertension [42]. These findings warrant further testing on FDA-approved or investigational drugs which possess the ability to alter host cell cholesterol levels or transport against the MKR.

## Materials and methods

### Cell and mycobacterial culture

Human monocytic THP-1 cells (ATCC TIB-202) were cultured in RPMI-1640 medium supplemented with 10% FBS, 4 mM *L*-glutamine, 10 mM HEPES, 1 mM sodium pyruvate, 4.5 g/L glucose and 0.05 mM 2-mercaptoethanol (Thermo Fisher Scientific) at 37°C and 5% CO_2_. *M. tuberculosis* reference strain H37Rv (BEI Resources; ATCC 25,618) and *M. tuberculosis* Beijing strain MKR superspreader (DS 5538) [3,15] were propagated in Middlebrook 7H9 medium supplemented with 0.05% Tween 80, 0.2% glycerol, and 10% oleic acid, albumin, dextrose, and catalase (BD Biosciences) at 37°C. The log-phase Mtb cultures were utilized in infection experiments and homogenized to generate a single-cell suspension. mCherry-expressing H37Rv and MKR [22,43] were grown in 7H9 medium, as described above, but supplemented with hygromycin (100 µg/mL; Invitrogen).

### Macrophage infection and total RNA extraction

THP-1 cells were differentiated into macrophages by plating into 6-well plates (1.5 x 10^6^ cells per well) and then treated with 100 nM of PMA (Sigma) for 24 hr. The cells were then washed and allowed to rest for 24 hr in complete media without PMA. Mtb infection was carried out by centrifuging the different mycobacteria onto macrophages at MOI of 20 (1,200 rpm for 5 min at room temperature) and then further incubated with the bacteria at 37°C and 5% CO_2_ for 30 min, as previously described [22,44]. The Mtb-infected cells were washed with RPMI1640 complete media three times to get rid of the uninternalized mycobacteria (T0, 0 hr post-infection) and were left in complete media for 4 hr (T4, 4 hr post-infection). At T0 and T4, the media were aspirated off and Trizol (ThermoFisher) was added. In addition, uninfected cells at T0 and T4 were used as controls and were also subjected to Trizol solubilization. Nucleic acids were then isolated from each sample as previously described [45]. The genomic DNAs were degraded using DNase (ThermoFisher). Subsequent total RNA isolation was done using the RNeasy kit (QIAGEN).

### Illumina library construction

The total RNA concentration of each sample was measured by using a DeNovix fluorometer (DeNovix). Nanodrop (Thermofisher) was used to determine the sample purity. The Agilent 2100 Bioanalyser (Agilent) was used to assess the integrity of the total RNAs. One microgram of the total RNAs from each sample was then subjected to QIAseq FastSelect RNA removal and QIAseq stranded total RNA library preparation kits (QIAGEN) to create individually-indexed, strand-specific RNA-Seq libraries. QIAseq FastSelect – rRNA HMR (Human, mouse, rat) removal solution (QIAGEN, USA) was used to get rid of the ribosomal RNA and the reaction was subjected to fragmentation and cDNA synthesis. cDNAs were then isolated using the AMPure XP beads (Beckman Coulter Genomics). cDNAs were then ligated with the indexing adapters and the quality of the cDNA libraries was inspected by using an Agilent 2100 Bioanalyser (Agilent) followed by quantification using a DeNovix fluorometer (DeNovix). Equimolar quantity of the indexed sequencing libraries was then subjected to cluster generation and paired-end 2 × 150 nucleotides read sequencing on an Illumina Hiseq sequencer.

### Differential gene expression analyses of RNA-Seq data

Trimmomatic V0.32 [46] was used to trim the adapters and eliminate the low-quality reads by applying the following parameters, ILLUMINACLIP: TruSeq3-PE.fa:2:30:10, LEADING:3, TRAILING:3, SLIDINGWI NDOW:4:15, and MINLEN:36. The residual high-quality reads were mapped to the human reference genome GRCh38 by using HISAT2 [47]. The featureCounts (Version 1.4.6) was then used to quantify the number of reads per transcript. Subsequently, DESeq2 V1.24.0 [48] was utilized to recognize DEGs between two experimental conditions. The Benjamini – Hochberg method was applied to adjust the *p*-values (≤0.05) for multiple test correction.

### Functional enrichment and network analyses

To ascertain significantly enriched ontology clusters under the default settings, DEGs were subjected to GO functional analysis using Metascape software [49]. Only clusters with enrichment scores of greater than 0.5 were considered. Moreover, the network analyses of the genes of interest were evaluated using GeneMANIA [50].

### RNA-Seq verification and qRT-PCR

Using random hexamers (Qiagen), 500 ng of total RNAs were reverse-transcribed. Primers used in this study were shown in the Supplementary Table S2 (Ward Medick). qRT-PCR analyses were then conducted using a thermocycler (Eco 48 Real-time PCR System, Illumina) with 2X PerfeCTa SYBR Green SuperMix (QuantaBio) and 0.2 mM forward and reverse primers at various annealing temperatures (54–66 °C). The specificity of the PCR products was verified by using the melting curve analyses. All threshold signals more than 95% efficient was obtained. The signals were normalized to the housekeeping *GAPDH* transcript and presented as relative quantification using the 2^*−*∆∆ct^ method.

### siRNA-mediated knockdown

In brief, THP-1 cells were harvested and re-suspended in 90 µL of human monocyte nucleofector solution (VPA-1007; Lonza). Scrambled siRNAs or siRNAs against *IL-36RN* (Dhamacon; 1.5 µg/reaction) were then added to the cells and the cell suspension was transferred into an electroporation cuvet and nucleofected using the Amaxa Nucleofector apparatus (Amaxa Biosystems) with programme Y-001, as previously described [44]. Cells were harvested and plated for assays at 24 hr after transfection.

### Intracellular Mtb survival assay

Intracellular Mtb survival assay was conducted by plating the THP-1 cells in 96-well black plates (7 x 10^4^ cells per well) and differentiated them into macrophages using 100 nM PMA, as described above. The macrophages were then infected with mCherry-expressing H37Rv or MKR at MOI of 10 by centrifuging Mtb onto the host cells (1,200 rpm for 5 min at room temperature) and then further incubated with the bacteria at 37°C and 5% CO_2_ for 1 hr as previously described [22,44]. The uninternalized mycobacteria were then removed by washing the cells with the complete media three times. Cells were further incubated in the complete media and at the indicated time points, cells were fixed with 4% paraformaldehyde for 30 min, stained with Hoechst for 15 min, and subjected to high-content image analysis (Operetta, PerkinElmer) to obtain the number of intracellular Mtb per cell [22,51]. Images were acquired from at least 7 different fields and more than 75 Mtb-infected cells were counted per well. Percent mycobacterial survival of each strain was then calculated relative to that of the 0 hr post-infection control set to 100%.

### Filipin III intracellular cholesterol staining

THP-1 cells were seeded in 96-well black plates (7 x 10^4^ cells per well) and differentiated into macrophages using 100 nM PMA, as described above. Cells were then infected with mCherry-expressing H37Rv or MKR or left uninfected for 1 hr. Cells were washed three times with complete media to get rid of the uninternalized mycobacteria. Cells were fixed at 24 hr post-infection with 4% paraformaldehyde for 15 min and then washed with PBS three times. To stain with Filipin III, stock solution (Sigma; 10 mg/mL) was diluted to 1:100 with 3% BSA in PBS and added to the cells for 2 hr at room temperature in the dark. Cells were then washed once with PBS and subjected to high content image analysis to determine the Filipin-III intensity per cell. Images were acquired from at least 7 different fields and more than 75 Mtb-infected cells were counted per well. Percent Filipin-III intensity per infected cell was then calculated and compared.

## Statistical analyses

All experiments were conducted at least three times. The data were pooled to compute the mean and standard errors of mean. All data were analysed by Prism 5.0 software (GraphPad). *p* values corrected for multiple testing of less than 0.05 were considered to indicate statistical significance.

## Supplementary Material

Supplemental Material

## Data Availability

The RNA-Seq data have been uploaded to the Gene Expression Omnibus (GEO) database (Accession number GSE194017).
