# Supplementary material for: Host cell transcriptomic response to the multidrug-resistant Mycobacterium tuberculosis clonal outbreak Beijing strain reveals its pathogenic features
Source: Virulence. 2022 Oct 15;13(1):1810–26. doi: 10.1080/21505594.2022.2135268 (PMC9578452; doi:10.1080/21505594.2022.2135268)
Supplement: Supplemental Material [file KVIR_A_2135268_SM1154.pdf]

Supplementary Information

Host cell transcriptomic response to the multidrug-resistant *Mycobacterium tuberculosis* clonal outbreak Beijing strain reveals its pathogenic features

Pinidphon Prombutara[a,b,c], Tegar Adriansyah Putra Siregar[c,d], Thanida Laopanupong[c], Phongthon Kanjanasirirat[e], Tanawadee Khumpanied[e], Suparerk Borwornpinyo[e,f], Awantika Rai[g], Angkana Chaiprasert[h,i], Prasit Palittapongarnpim[c,j,k], Marisa Ponpuak[c, j*]

Supplementary Methods

CFU analysis

CFU analysis was conducted by plating the THP-1 cells in 96-well plates (7 x 10^4 cells per well) and differentiated them into macrophages using 100 nM PMA, as described in the Materials and Methods. The macrophages were then infected with H37Rv or MKR at MOI of 10 by centrifuging Mtb onto the host cells (1,200 rpm for 5 min at room temperature) and then further incubated with the bacteria at 37°C and 5% CO_2 for 1 hr. The uninternalized mycobacteria were then removed by washing the cells with the complete media three times. Cells were further incubated in the complete media and at the indicated time points, cells were then lyzed by osmotic burst to harvest the intracellular mycobacteria followed by serial dilution and plated onto the Middlebrook 7H10 agars containing 10% OADC, 0.2% glycerol and 0.05% Tween 80. Plates were incubated at 37°C and colonies were counted. Percent mycobacterial survival was then calculated relative to that of the respective control set to 100%.

In vitro growth analysis

For in vitro growth analysis, each Mtb strain was cultured in the Middlebrook 7H9 medium containing 10% OADC, 0.2% glycerol and 0.05% Tween 80 at 37 °C. At day 0, the cultures were diluted to OD600 = 0.01 and thereafter the OD600 values were measured daily.

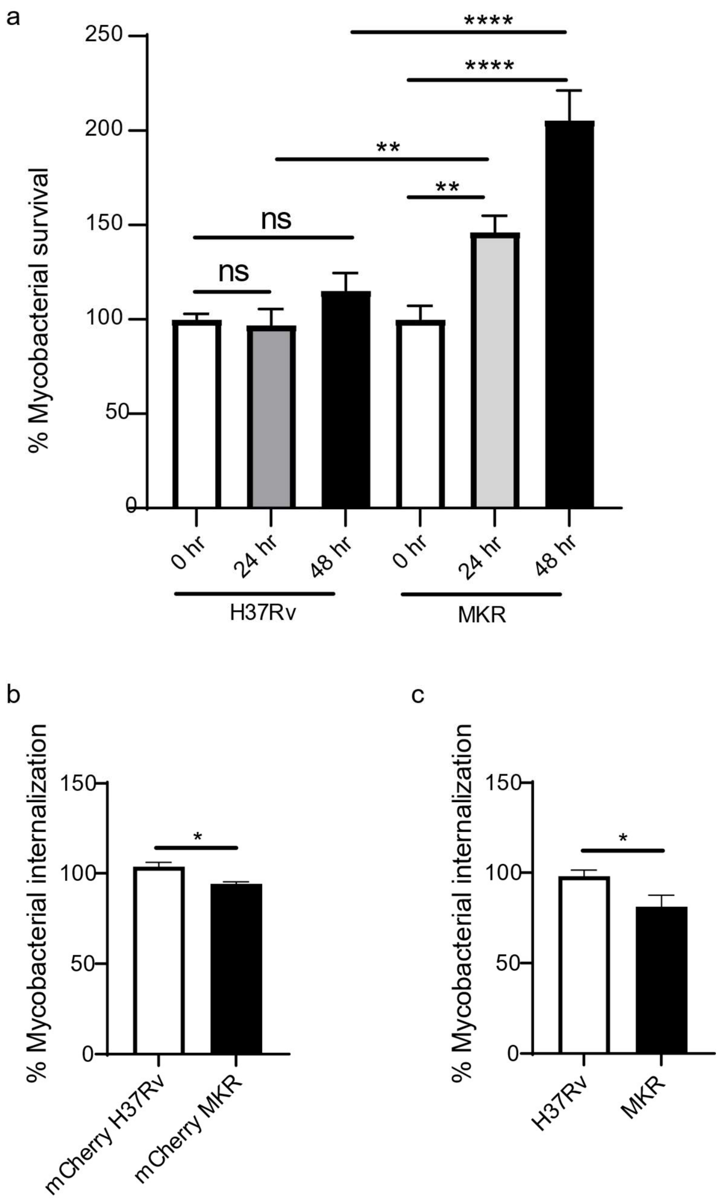

Supplementary Figure 1. Internalized rates of the MKR superspreader and H37Rv into host macrophages and CFU analysis. (a) THP-1 cells were infected with the MKR superspreader or H37Rv for 1 hr. Cells were washed to eliminate the uninternalized mycobacteria. At 0, 24, and 48 hr after infection, cells were lyzed by osmotic burst to harvest intracellular mycobacteria. Number of intracellular mycobacteria was then determined by plating for CFU and percent mycobacterial survival was calculated. Data are means ± SEM from at least three independent experiments; ns, non-significant, **$p<0.01$ and ****$p < 0.0001$, all relative to the respective 0 hr control set to 100%, were determined by one-way ANOVA with Tukey's multiple comparison test. **(b)** THP-1 cells were infected with mCherry-expressing MKR superspreader or H37Rv for 1 hr. Cells were washed to eliminate the uninternalized

mycobacteria and fixed (at 0 hr after infection) for high-content image analysis to determine the internalized mycobacterial number per cell. Percent mycobacterial internalization was then calculated. Data are means ± SEM from at least three independent experiments; *p < 0.05, relative to the mCherry H37Rv-infected cells set to 100% was determined by Student's t-test. **(c)** THP-1 cells were infected with the MKR superspreader or H37Rv for 1 hr. Cells were washed to eliminate the uninternalized mycobacteria (at 0 hr after infection) and lyzed by osmotic burst to harvest intracellular mycobacteria. Number of internalized mycobacteria was then determined by plating for CFU. Percent mycobacterial internalization was then calculated. Data are means ± SEM from at least three independent experiments; *p < 0.05, relative to the H37Rv-infected cells set to 100% was determined by Student's t-test.

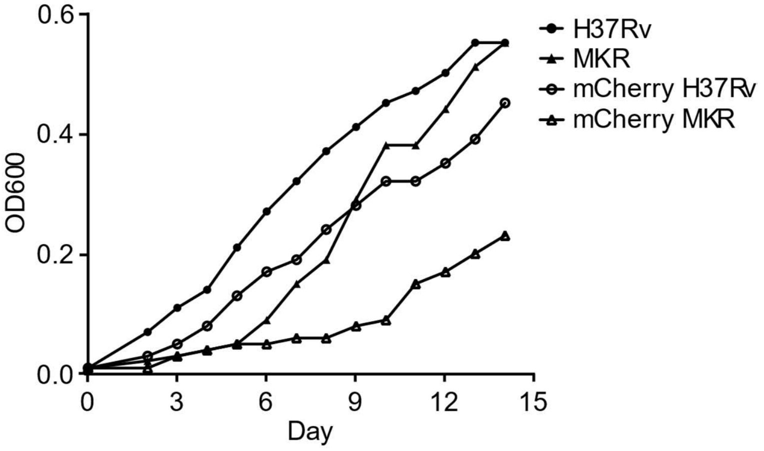

Supplementary Figure 2. In vitro growth rates of the MKR superspreader and H37Rv. The MKR and H37Rv were grown in 7H9 media containing 10% OADC, 0.2% glycerol and 0.05% Tween 80 at 37 °C for 14 days. The mCherry-expressing MKR and H37Rv were grown in 7H9 media as described above, supplemented with 100 µg/mL hygromycin. At day 0, the cultures were diluted to OD600 = 0.01 in the respective media. The OD600 values were then recorded daily for the mycobacterial cultures.

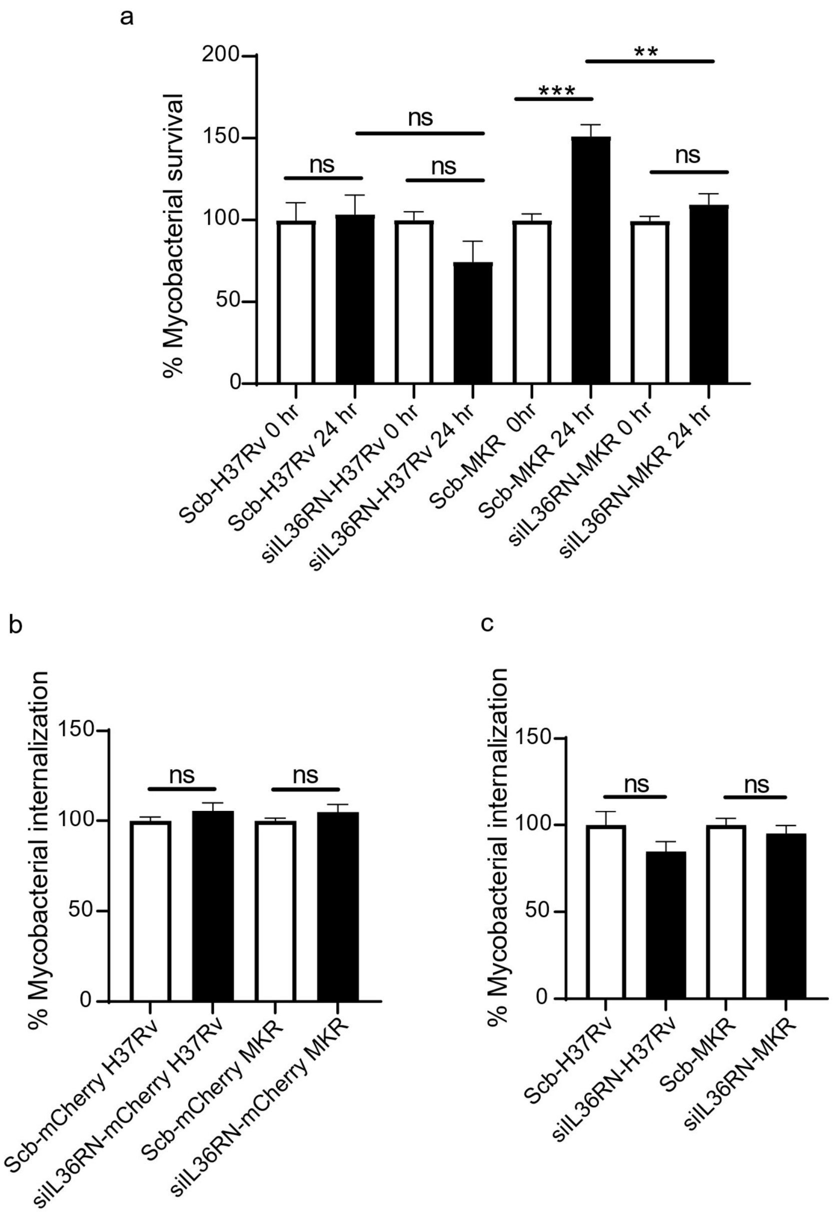

Supplementary Figure 3. Internalized rates of the MKR superspreader and H37Rv into IL-36RN-deficient macrophages and CFU analysis. (a) THP-1 cells were transfected with the non-targeted scrambled control (Scb) or *IL-36RN*-targeted siRNAs. At 48 hr after transfection, THP-1 cells were infected with the MKR superspreader or H37Rv for 1 hr. Cells were washed to eliminate the uninternalized mycobacteria. At the indicated time points, cells were lyzed by osmotic burst to determine the number of intracellular mycobacteria by plating for CFU. Percent mycobacterial survival was then calculated. Data are means ± SEM from at least three independent experiments; ns, non-significant, **$p<0.01$ and ***$p < 0.001$, all relative to the respective 0 hr control set to 100%, were determined by one-way ANOVA with Tukey's multiple comparison test. **(b)** siRNA-transfected THP-1 cells were infected with the

mCherry-expressing MKR or H37Rv for 1 hr. Cells were washed to eliminate the uninternalized mycobacteria and fixed (at 0 hr after infection) for high-content image analysis to determine the internalized mycobacterial number per cell. Percent mycobacterial internalization was then calculated. Data are means ± SEM from at least three independent experiments; ns, non-significant, all relative to the respective scrambled siRNA control cells set to 100%, was determined by one-way ANOVA with Tukey's multiple comparison test. **(c)** siRNA-transfected THP-1 cells were infected with the MKR or H37Rv as in (a). At 0 hr post-infection time point, cells were then lyzed by osmotic burst to determine the number of internalized mycobacteria by plating for CFU. Percent mycobacterial internalization was then calculated. Data are means ± SEM from at least three independent experiments; ns, non-significant, all relative to the respective scrambled siRNA control cells set to 100%, was determined by one-way ANOVA with Tukey's multiple comparison test.

Supplementary Table 1. Summary of the DEGs between conditions.

Condition	H37Rv (T4 vs T0)	MKR (T4 vs T0)
**Up-regulated**	2,321	2,103
**Down-regulated**	2,873	2,752
**Total**	5,194	4,855

Supplementary Table 2. Primer sequences used for qRT-PCR in this study.

Gene name	Primer name	Sequence (5' – 3')
ALKAL1	ALKAL1_F1	5′-GAGTGCTCAACGCCAGCTTA-3′
	ALKAL1_R1	5′-AGTTTTGCTAGGTCTGGGAGC-3′
MUC20	MUC20_F1	5′-CAAGATCACAACCTCAGCGA-3′
	MUC20_R1	5′-ACCTCCATTTTCACCTGCAC-3′
MAP3K14	MAP3K14_F1	5′-ACTTTGGCCATGCTGTGTGTC-3′
	MAP3K14_R1	5′-GGTCTCTGTGCCAGGGATGTAG-3′
NUPR1	NUPR1_F1	5′-GCGGGCACGAGAGGAAAC-3′
	NUPR1_R1	5′-CTCAGTCAGCGGGAATAAGTC-3′
RAB42	RAB42_F1	5′-GGTGCATCACCAGGTCCTTT-3′
	RAB42_R1	5′-GGAAGATGACCTTGTCCGGG-3′
RGL3	RGL3_F1	5′-CCTTGCAGAAGCACAATGTGC-3′
	RGL3_R1	5′-CGTTGGCATTGTCAGGAATCA-3′
SMIM25	SMIM25_F1	5′-GATCCTAGAGGAAAGTGGCAAG-3′
	SMIM25_R1	5′-GGGTGTACAGCAGTGAACAA-3′
SNAI1	SNAI1_F1	5′-CCTCAAGATGCACATCCGAAG-3′
	SNAI1_R1	5′-ACATGGCCTTGTAGCAGCCA-3′
TRAF2	TRAF2_F1	5′-GGCGATGGCTGACTTGGA-3′
	TRAF2_R1	5′-CTGCTGGTGTAGAAGGCTGG-3′
TRIM63	TRIM63_F1	5′-GGAGCCACCTTCCTCTTGAC-3′
	TRIM63_R1	5′-GTCAATGGCTCTCAGGGCGT-3′
PMAIP1	PMAIP1_F1	5′-AGCTGGAAGTCGAGTGTGCT-3′
	PMAIP1_R1	5′-TCCTGAGCAGAAGAGTTTGGA-3′
EDN1	EDN1_F1	5′-TGGGAAAAAGTGTATTTATCAGCA-3′
	EDN1_R1	5′-TTTGACGCTGTTTCTCATGG-3′
IL-36RN	IL-36RN_F1	5′-GGAGAAAGGAACATTCTGAGGG-3′
	IL-36RN_R1	5′-GGGACCACGCTGATCTCTT-3′
IL-36G	IL-36G_F1	5′-GAAGTGACAGTGTGACCCCA-3′
	IL-36G_R1	5′-CGGGTTGGCCATACAGATCC-3′
